# Serum complement C1q level is associated with left ventricular hypertrophy induced by coarctation of the aorta: A retrospective observational study

**DOI:** 10.1186/s12872-022-02807-2

**Published:** 2022-08-10

**Authors:** Li Chen, Hong-zhou Duan, Chen Zhang, Gang Li, Yong-tao Wu, Dong Wang, Xiao-yan Li

**Affiliations:** 1 Department of Pediatric Cardiology, Beijing Anzhen Hospital, Capital Medical University, Beijing, China; 2 Department of Neurosurgery, Peking University First Hospital, Beijing, China; 3 Department of Pediatric Cardiac Surgery, Beijing Anzhen Hospital, Capital Medical University, Beijing, China; 4 Beijing Institute of Heart, Lung and Blood Vessel Diseases, Capital Medical University, No.2 Anzhen Street, Chaoyang District, Beijing, 100029 China

**Keywords:** Aortic coarctation, Complement C1q, Left ventricular hypertrophy, Myocardial injury, Lipid metabolism

## Abstract

**Background:**

The complement system plays an important role in the development of left ventricular hypertrophy. Complement C1q is an initial component of the classical complement pathway and is related to many inflammatory diseases. We aimed to determine whether there was an association between serum complement C1q and left ventricular hypertrophy induced by coarctation of the aorta (CoA).

**Methods:**

Based on whether CoA was combined with a large ventricular septal defect (VSD) or patent ductus arteriosus (PDA), the patients were divided into a simple CoA group (*n* = 15) and a complex CoA group (*n* = 13). Meanwhile, we selected simple large VSD (*n* = 14) patients and normal children (*n* = 28) as the control group. The serum complement C1q level was compared using immunity transmission turbidity among different groups.

**Results:**

The preoperative content of C1q in the simple CoA group was significantly lower than that in the complex CoA group and normal group (96.97 ± 20.66 vs. 130.73 ± 35.78, 96.97 ± 20.66 vs. 156.21 ± 29.14, *P* < 0.05). There was no significant difference in the preoperative content of C1q between the complex CoA group and the large VSD group (*P* > 0.05). There was a negative correlation between the preoperative complement C1q content and the interventricular septal thickness and left ventricular posterior wall thickness (*r* = − 0.035, *r* = − 0.288, *P* < 0.05). The percentage of postoperative decrease in C1q in children with simple CoA or complex CoA was positively correlated with the time of cardiopulmonary bypass and aortic cross clamp, respectively (*r* = 0.797, *r* = 0.622, *r* = 0.898, *r* = 0.920, *P* < 0.05). There was no significant difference in the content of preoperative triglycerides (TG), total cholesterol (TCHO), high-density lipoprotein cholesterol (HDL-C) or low-density lipoprotein cholesterol (LDL-C) among the different groups (*P* > 0.05). In the simple CoA group and complex CoA group, the preoperative complement C1q, TG, TCHO, HDL-C and LDL-C levels were significantly higher than those after the operation (*P* < 0.05). There was no significant correlation between preoperative complement C1q and TG, TCHO, HDL-C or LDL-C (*P* > 0.05).

**Conclusions:**

Complement C1q has an inhibitory effect on the formation of left ventricular hypertrophy, which may not be mediated by regulating lipid metabolism. During cardiac surgery, complement C1q may have a protective effect against myocardial injury.

## Background

Congenital coarctation of the aorta (CoA) is a common congenital heart disease. Its incidence is approximately 1/2500 live birth infants, and it accounts for 6 ~ 8% [1] of all congenital heart diseases. CoA may be seen in isolation or with other congenital heart anomalies, such as bicuspid aortic valve, ventricular septal defect, patent ductus arteriosus, transposition of the great arteries, atrioventricular canal defects, or left­sided obstructive heart defects [2–4]. According to whether it is isolated or combined with other congenital heart anomalies, CoA can be classified as isolated/simple CoA and complex CoA [5]. Some simple CoA may have no obvious symptoms in the early stage, which is easy to ignore and escapes diagnosis. If it is not treated in time, patients usually die of complications such as heart failure, aortic rupture or intracranial hemorrhage due to hypertension [6]. Refractory hypertension and blood pressure differences between the upper and lower limbs are the most important clinical manifestations and signs of CoA [6]. CoA can increase the left ventricular afterload, leading to coarctation proximal hypertension [7]. The increase in the left ventricular afterload will lead to changes in the myocardial tissue and ventricular structure [8, 9]. At the cellular level, it will cause activation and pathological proliferation of cardiomyocyte interstitial fibroblasts, which will lead to left ventricular myocardial fibrosis, hypertrophy and congestive heart failure [10].

The complement system is an essential part of innate and adaptive immunity. It comprises of a group of proteins with enzyme activity in the blood, body fluids and on the surface of the cell membrane. It plays a biological function in regulating phagocytosis, clearing aging apoptotic cells, participating in the immune response and mediating the inflammatory response [11]. C1q is an important promoter of the classical complement pathway. It activates the complement cascade reaction by recognizing the complement binding site of the antibody FC segment in an IgG or IgM immune complex, which leads to clearance of the antigen antibody complex [12]. The complement C1q tumor necrosis factor related protein (CTRPs) superfamily is a cluster of adipokines. The family consists of 15 members. They have been proven to have diverse biological influences on the cardiovascular system [13]. Some studies reported that CTRP-3 could attenuate pressure overload-induced cardiac hypertrophy [14] and the CTRP-6 could attenuate postinfarct cardiac fibrosis [15]. The structure of CTRPs is similar to that of the complement component C1q. Some studies have shown that complement C1q can induce skeletal muscle fibrosis by regulating the Wnt/*β*-Catenin pathway [16].Therefore, we wondered whether complement C1q is involved in left ventricular hypertrophy. It has not been reported until now.

Therefore, determining the level of complement C1q in patients with CoA will be of great significance to further clarify the pathogenesis of left ventricular hypertrophy induced by CoA. In this study, we analyzed the content of complement C1q and the changes in complement C1q before and after surgery in patients with CoA and clarified the regulatory effect of complement C1q on CoA-induced left ventricular hypertrophy and the correlation between operation-related factors and complement C1q.

## Methods

### Materials

Patients with CoA hospitalized in the pediatric heart center of Beijing Anzhen Hospital between January 1, 2017 and December 31, 2019 were included in this study. Inclusion criteria: (1) Meets the diagnostic criteria of CoA; (2) Clinical diagnosis of simple CoA or complex CoA (CoA with a large ventricular septal defect (VSD) (defect diameter ≥ 10 mm) or CoA with a thick patent ductus arteriosus (PDA) (inner diameter of pulmonary end of arterial catheter ≥ 5 mm), and a left to right shunt at ventricular level or large artery level); (3) Complement C1q examination was performed before and 24 h after the operation, and the clinical data were complete; (4) There were no other systemic diseases. Exclusion criteria: (1) patients with CoA who did not undergo surgery; (2) patients with an early death; (3) CoA with a small ventricular septal defect (defect diameter < 5 mm) or a small patent ductus arteriosus (pulmonary end of the ductus arteriosus < 2 mm), or CoA with other cardiovascular malformations; (4) CoA with a large ventricular septal defect or a thick patent ductus arteriosus and a right to left shunt at the ventricular or large artery level; (5) combined with other systemic diseases; (6) incomplete clinical data before and after the operation. Meanwhile, we selected children with simple large VSD with complete clinical data (defect diameter ≥ 10 mm, left to right shunt at the ventricular level) and normal children as controls. The institutional review board for clinical research at Beijing Anzhen Hospital approved the use of patient medical records for this retrospective review.

### Methods

#### 2.2.1 Group division

Based on whether CoA patients had a large VSD or PDA, the patients were divided into a simple CoA group and a complex CoA group (CoA combined with left to right shunt congenital heart disease). The control group was divided into a simple large VSD group and normal group.

#### 2.2.2 Measurement of echocardiographic parameters

All patients were measured by transthoracic echocardiography with an ultrasonic instrument (PHILIP IE33) one day before the operation. The position and length of the aortic constriction were measured by two-dimensional echocardiography through the long axis section of the suprasternal aortic arch, and the blood flow velocity at the constriction was measured by color Doppler flow imaging. Left ventricular end diastolic diameter (LVDd), interventricular septal thickness (IVST) and left ventricular posterior wall thickness (LVPWT) were measured by M-mode echocardiography through the parasternal left ventricular long axis section. The diameter of the VSD and the left to right shunt velocity at the ventricular level were measured through multiple sections by two-dimensional echocardiography and color Doppler flow imaging. The diameter, length and left to right shunt velocity of the patent ductus arteriosus were measured by two-dimensional echocardiography and color Doppler flow imaging through the short axis section of the parasternal artery.

IVST, LVPWT and LVDd were converted to Z scores (number of standard deviations from the expected mean) using the formula from Sluymans and Colan [17].

#### 2.2.3 Measurement of complement C1q

Venous blood was taken from patients with an empty stomach in the case group one day before the operation and 24 h after the operation in the morning. Venous blood was taken from the children in the control group with an empty stomach in the morning. The serum was separated from the blood samples. The content of complement C1q was determined by a complement C1q determination kit (immunoturbidimetry) (Shanghai Beijia Biochemical Reagent Co., Ltd., China).

#### 2.2.4 Measurement of triglyceride (TG), total cholesterol (TCHO), high-density lipoprotein cholesterol (HDL-C) and low-density lipoprotein cholesterol (LDL-C)

The venous blood of patients with an empty stomach was taken before the operation, and serum samples were used to determine the contents of TG, TCHO, HDL-C and LDL-C.

#### 2.2.5 Patients with simple or complex CoA were all treated with surgery.

The intraoperative cardiopulmonary bypass time, aortic cross clamp time and mechanical ventilation time were collected.

### Statistical analysis

SPSS 20.0 was used for statistical analysis. Measurement data are presented as the mean ± standard deviation ($\overline{X} \pm S$), and count data are expressed as the frequency (percentage). The *χ2* test was performed to analyze the sex distribution. A *t*-test was used to compare the differences between the two groups of normally distributed measurement data, and one-way ANOVA was used to compare the differences among three or more groups. The Mann–Whitney *U* test was used to compare the nonnormally distributed measurement data between the two groups. For the correlation between complement C1q and IVSTZ, LVPWTZ, LVDdZ, TG, TCHO, HDL-C, LDL-C, cardiopulmonary bypass time, aortic cross clamp time and mechanical ventilation time, if the data conformed to a normal distribution, pearson correlation analysis was used; if the data did not conform to a normal distribution, rank correlation analysis was used. *P* < 0.05 was considered statistically significant.

## Results

### Clinical characteristics

There were 28 cases in the case group, including 15 cases (53.6%) in the simple CoA group and 13 cases (46.4%) in the complex CoA group (11 cases (39.3%) were CoA combined with large VSD, and 2 cases (7.1%) were CoA combined with thick PDA). There were 42 cases in the control group, including 14 cases (33.3%) in the simple large VSD group and 28 cases (66.7%) in the normal group.

There was no significant difference in sex, age, weight or height among the simple CoA group, complex CoA group, simple large VSD group and normal group (P > 0.05) (Table 1).

**Table 1:** The basic information of patients in different groups

Group	*n*	Sex				
		Male	Female	Age (Month)	Weight (kg)	Height (cm)
Simple CoA	15	9	6	5.87 ± 2.17	5.70 ± 1.36	63.33 ± 5.89
Complex CoA	13	9	4	7.62 ± 5.03	6.36 ± 1.85	67.38 ± 7.58
Normal	28	18	10	6.75 ± 2.35	6.49 ± 0.97	66.46 ± 5.05
Large VSD	14	9	5	7.71 ± 2.61	6.02 ± 1.22	66.43 ± 5.05
*X*^*2*^		0.258				
*F*				1.181	1.367	1.404
*P*		0.968	0.324	0.261	0.249	

*CoA* Coarctation of the aorta, *VSD* Ventricular septal defect

### The differences in IVSTZ, LVPWTZ and LVDdZ in different groups

The IVSTZ and LVPWTZ of patients in the simple CoA group were significantly higher than those in the complex CoA group and normal group (*P* < 0.05). There was no significant difference in LVDdZ between the complex CoA group and the normal group (*P* > 0.05). There was no significant difference in IVSTZ and LVPWTZ between patients in the complex CoA group and the large VSD group (*P* > 0.05), but the LVDdZ in the complex CoA group was significantly lower than that in the large VSD group (*P* < 0.05) (Table 2).

**Table 2:** The IVSTZ, LVPWTZ and LVDdZ of patients in different groups

Group	*n*	IVSTZ median (minimum, maximum)	LVPWTZ median (minimum, maximum)	LVDdZ median (minimum, maximum)
Simple CoA	15	2.46 (0.29, 8.71)	2.36 (0.52, 10.62)	2.66 (− 1.29, 4.89)
Complex CoA	13	1.00 (0.14, 5.86)	0.76 (-0.15, 1.98)	2.36 (1.06, 5.77)
Normal	28	0.26 (− 1.37, 1.79)	0.52 (− 1.07, 1.74)	1.77 (− 0.35, 5.03)
Large VSD	14	0.84 (0.14, 2.52)	0.74 (− 0.15, 2.18)	3.96 (2.04, 6.39)
*Z1*		− 2.699	− 3.017	− 0.530
*P*		0.007	0.003	0.596
*Z2*		− 4.167	− 4.422	− 1.147
*P*		0.000	0.000	0.251
*Z3*		− 0.267	− 0.51	− 2.209
*P*		0.789	0.610	0.027

*CoA* Coarctation of the aorta *VSD* Ventricular septal defect, *Z1* Simple CoA group vs. Complex CoA group, Z2: Simple CoA group vs. Small VSD group, Z3: Complex CoA group vs. Large VSD group, IVSTZ: interventricular septal thickness Z score, LVPWTZ: left ventricular posterior wall thickness Z score, LVDdZ: left ventricular end diastolic diameter Z score

### TG, TCHO, HDL-C and LDL-C values and their correlation with complement C1q in different groups

**Fig. 1:**
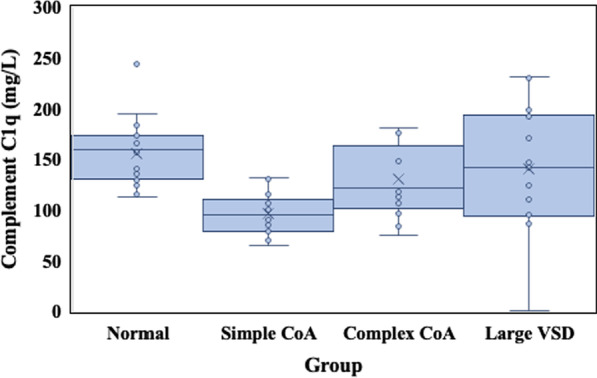
The distribution of preoperative content of complement C1q in normal group, simple CoA group, complex CoA group and large VSD group. (The preoperative content of complement C1q in the simple CoA group (*n* = 15) was significantly lower than that in the complex CoA group (*n* = 13) and normal group (*n* = 28) (*P* < 0.05); There was no significant difference in preoperative complement C1q content between complex CoA group (*n* = 13) and large VSD group (*n* = 14) (*P* > 0.05).)

The preoperative content of complement C1q in the simple CoA group was significantly lower than that in the complex CoA group and normal group (96.97 ± 20.66 vs. 130.73 ± 35.78, 96.97 ± 20.66 vs. 156.21 ± 29.14, t = 3.109, t = − 6.973, *P* < 0.05) (Fig. 1). There was no significant difference in preoperative complement C1q content between the complex CoA group and the large VSD group (130.73 ± 35.78 vs. 147.62 ± 50.38, t = − 0.997, *P* > 0.05) (Fig. 1). The postoperative content of complement C1q in the simple CoA group or complex CoA group was significantly lower than it was pre-operation (69.40 ± 24.19 vs 96.97 ± 20.66, 78.38 ± 28.12 vs. 130.73 ± 35.78, t = 6.853, t = 5.914, *P* < 0.05) (Table 3).

**Table 3:** The preoperative and postoperative C1q values of patients in children with CoA

Group	*n*	Complement C1q (mg/L)	Complement C1q (mg/L)	*t*	*P*
		Pre-operation	Post-operation		
Simple CoA	15	96.97 ± 20.66	69.40 ± 24.19	6.853	0.000
Complex CoA	13	130.73 ± 35.78	78.38 ± 28.12	5.914	0.000

There was no significant difference in the contents of TG, TCHO, HDL-C and LDL-C before operation in each group (*P* > 0.05) (Table 4). In simple CoA group and complex CoA group, the preoperative complement C1q, TG, TCHO, HDL-C and LDL-C levels were significantly higher than those after the operation, respectively (*P* < 0.05) (Table 4). There was no significant correlation between preoperative complement C1q and TG, TCHO, HDL-C or LDL-C (*P* > 0.05) (Table 4). In the simple CoA group, there was no significant correlation between postoperative complement C1q and TG, TCHO, HDL-C or LDL-C (*P* > 0.05) (Table 4).However, in the complex CoA group, postoperative complement C1q was positively correlated with TCHO, HDL and LDL (*r* = 0.893, 0.929, 0.819, *P* < 0.05) (Table 4).

**Table 4:** The C1q, TG, TCHO, HDL-C and LDL-C values of patients in different groups

Group		n	Complement C1q (mg/L)	TG (mmol/L)	TCHO (mmol/L)	HDL (mmol/L)	LDL (mmol/L)
Simple CoA	Pre-operation	15	96.97 ± 20.66	1.15 ± 0.45	4.04 ± 0.74	1.23 ± 0.33	2.31 ± 0.60
	Post-operation	15	69.40 ± 24.19	0.67 ± 0.36	2.65 ± 0.75	0.88 ± 0.31	1.35 ± 0.51
	*t*		6.853	4.835	5.180	3.862	5.021
	*P*		0.000	0.000	0.000	0.002	0.000
	*r*			0.006	− 0.148	− 0.062	− 0.046
	*P*			0.982	0.598	0.826	0.870
	*r1*			0.308	0.469	0.276	0.482
	*P1*			0.264	0.078	0.319	0.069
Complex CoA	Pre-operation	13	130.73 ± 35.78	1.33 ± 0.67	3.82 ± 0.77	1.14 ± 0.30	2.33 ± 0.58
	Post-operation	13	78.38 ± 28.12	0.59 ± 0.27	2.26 ± 1.04	0.79 ± 0.42	1.10 ± 0.66
	*t*		5.914	4.259	6.051	2.676	8.360
	*P*		0.000	0.001	0.000	0.020	0.000
	*r*			0.099	0.254	− 0.397	0.420
	*P*			0.747	0.403	0.179	0.153
	*r1*			0.084	0.893	0.929	0.819
	*P1*			0.785	0.000	0.000	0.001
*t1*			0.908	− 0.645	− 1.139	− 0.683	− 1.153
*P1*			0.372	0.525	0.265	0.501	0.260
Normal		28	156.21 ± 29.14	1.23 ± 0.85	3.98 ± 0.61	1.14 ± 0.29	2.30 ± 0.44
	*r*			− 0.109	− 0.209	− 0.193	− 0.095
	*P*			0.5579	0.286	0.325	0.629
Large VSD		14	147.62 ± 50.38	1.52 ± 0.99	4.12 ± 0.63	1.19 ± 0.28	2.36 ± 0.52
	*r*			0.302	0.052	0.010	0.068
	*P*			0.294	0.860	0.973	0.817
*F*			10.381	0.610	0.489	0.380	0.042
*P*			0.000	0.611	0.691	0.768	0.988

CoA: Coarctation of the aorta, VSD: Ventricular septal defect, TG: triglyceride, TCHO: total cholesterol, HDL: high density lipoprotein, LDL: low density lipoprotein, t stands for preoperative C1q, TG, TCHO, HDL and LDL compared to post-operation in simple CoA group or complex CoA group, t1 stands for postoperative simple CoA group compared to complex CoA group, r stands for the correlation between preoperative complement C1q and preoperative TG, TCHO, HDL-C and LDL-C, r1 stands for the correlation between postoperative complement C1q and postoperative TG, TCHO, HDL-C and LDL-C. F stands for preoperative C1q, TG, TCHO, HDL and LDL comparation among simple CoA group, complex CoA group, normal group and large VSD group

### The correlation between complement C1q and left ventricular hypertrophy

**Fig. 2:**
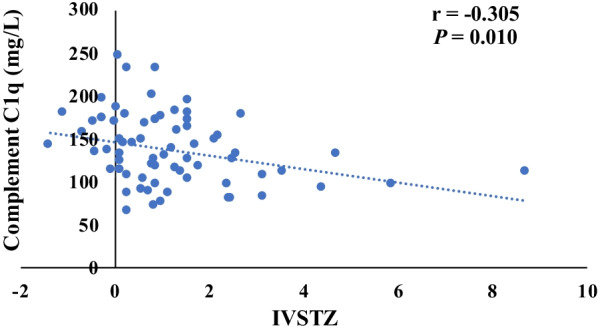
The correlation of complement C1q and IVSTZ. (The content of preoperative complement C1q was negatively correlated with the IVSTZ (*n* = 70, *r* = − 0.035, *P* < 0.05).)

**Fig. 3:**
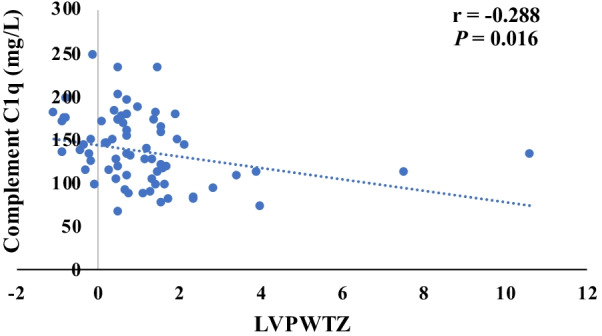
The correlation of complement C1q and LVPWTZ (The content of preoperative complement C1q was negatively correlated with the LVPWTZ (*n* = 70, *r* = − 0.288, *P* < 0.05).)

**Fig. 4:**
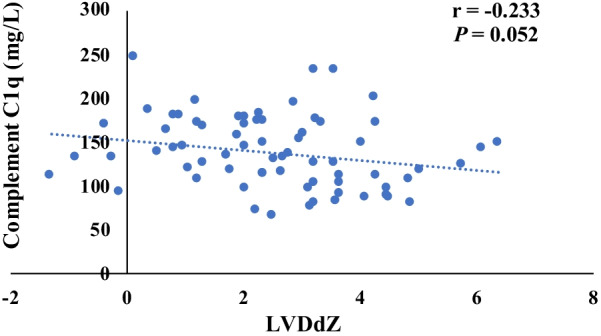
The correlation of complement C1q and LVDdZ (There was no significant correlation between the content of preoperative complement C1q and LVDdZ (*n* = 70, *r* = − 0.233, *P* > 0.05).)

The content of preoperative complement C1q was negatively correlated with the IVSTZ and LVPWTZ (*r* = − 0.035, *r* = − 0.288, *P* < 0.05) (Fig. 2, Fig. 3), and there was no significant correlation with LVDdZ (*r* = − 0.233, *P* > 0.05) (Fig. 4).

### The correlation between complement C1q and intraoperative related factors

Patients in the simple CoA group and complex CoA group were treated with surgery. In the simple CoA group, 4 children did not undergo cardiopulmonary bypass during the operation, and in the complex CoA group, 5 children did not undergo cardiopulmonary bypass. There was no significant difference in the percentage of postoperative complement C1q decrease between the simple CoA group and the complex CoA group (0.40 ± 0.17 vs. 0.36 ± 0.21, *t* = 0.515, *P* > 0.05). There was no significant difference in cardiopulmonary bypass time, aortic cross clamp time or mechanical ventilation time between the simple CoA group and the complex CoA group (*P* > 0.05) (Table 5).

**Table 5:** The CPB time, aortic cross clamp time and mechanical ventilation time in children with CoA

Parameter	Simple CoA group	Complex CoA group	*t*	*P*
N	11	8		
The percentage of postoperative complement C1q decrease	0.40 ± 0.17	0.36 ± 0.21	0.515	0.613
CPB time (min)	136.55 ± 29.73	135.00 ± 46.96	0.088	0.931
*r*	0.797	0.898		
*P*	0.003	0.002		
Aortic cross clamp time (min)	67.27 ± 14.06	69.13 ± 26.54	− 0.198	0.846
*r*	0.622	0.920		
*P*	0.041	0.001		
Mechanical ventilation time	78.13 ± 37.58	78.56 ± 35.02	− 0.025	0.980
*r*	0.620	0.699		
*P*	0.042	0.054		

CoA: Coarctation of the aorta, CPB: Cardiopulmonary bypass

**Fig. 5:**
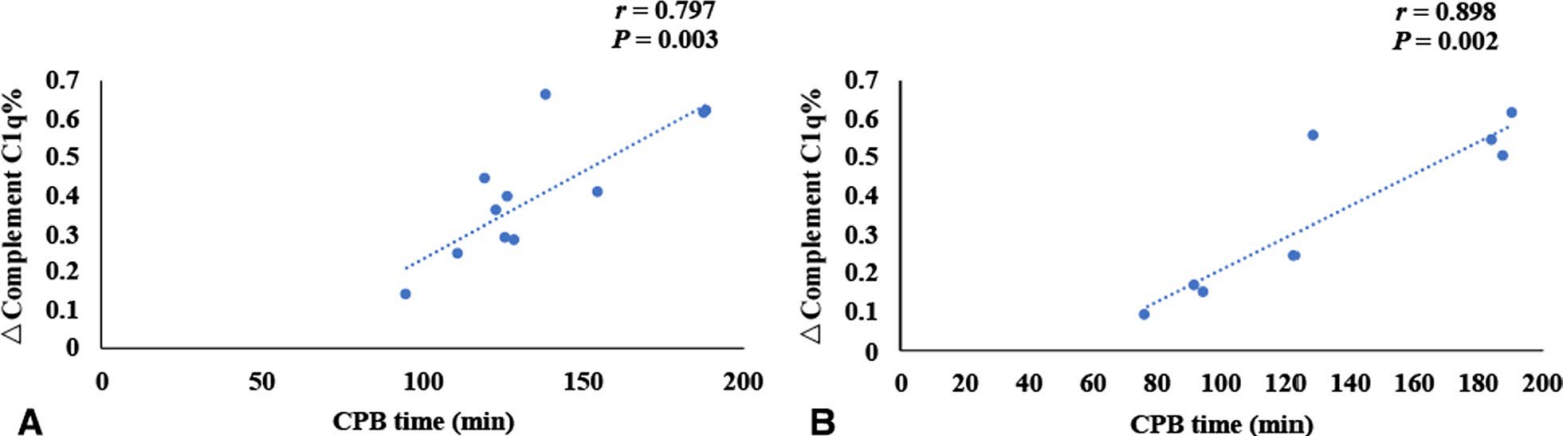
The correlation of CPB time and the percentage decrease of complement C1q before and after operation in simple CoA group and complex CoA group. **A** shows the correlation of CPB time and the percentage decrease of complement C1q before and after operation in simple CoA group (*n* = 11); **B** shows the correlation of CPB time and the percentage decrease of complement C1q before and after operation in complex CoA group (*n* = 8). The percentage of postoperative decrease in C1q in children with simple CoA or complex CoA was positively correlated with the time of cardiopulmonary bypass (CPB), respectively (*r* = 0.797, *r* = 0.898, *P* < 0.05).)

**Fig. 6:**
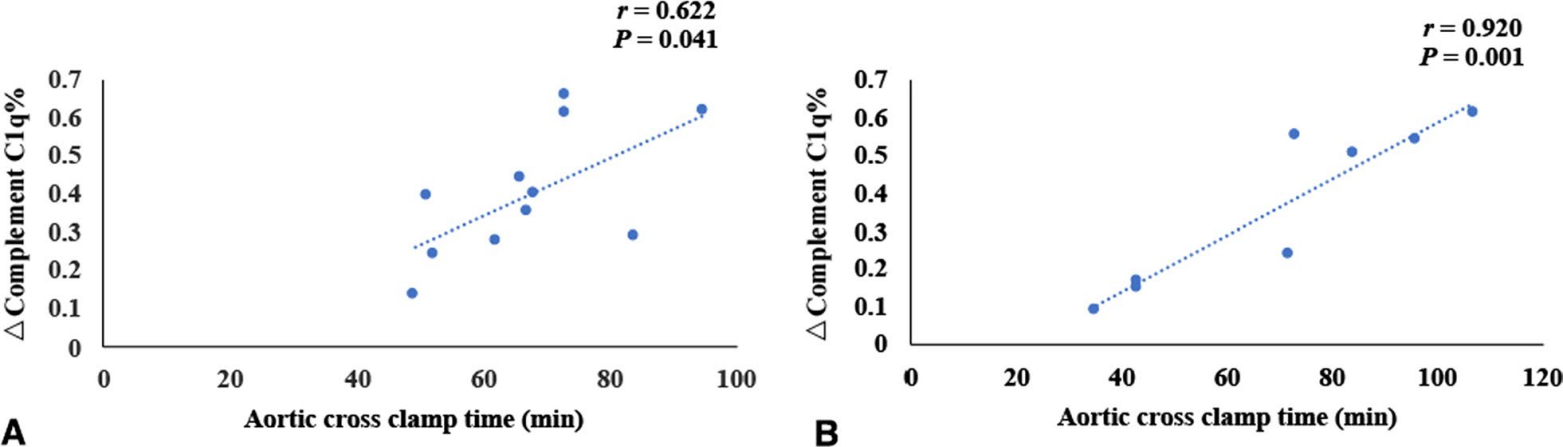
The correlation of aortic cross clamp time and the percentage decrease of complement C1q before and after operation in simple CoA group and complex CoA group. (**A** shows the correlation of aortic cross clamp time and the percentage decrease of complement C1q before and after operation in simple CoA group (*n* = 11); **B** shows the correlation of aortic cross clamp time and the percentage decrease of complement C1q before and after operation in complex CoA group (*n* = 8). The percentage of postoperative decrease in C1q in children with simple CoA or complex CoA was positively correlated with aortic cross clamp time, respectively (*r* = 0.622, *r* = 0.920, *P* < 0.05).)

**Fig. 7:**
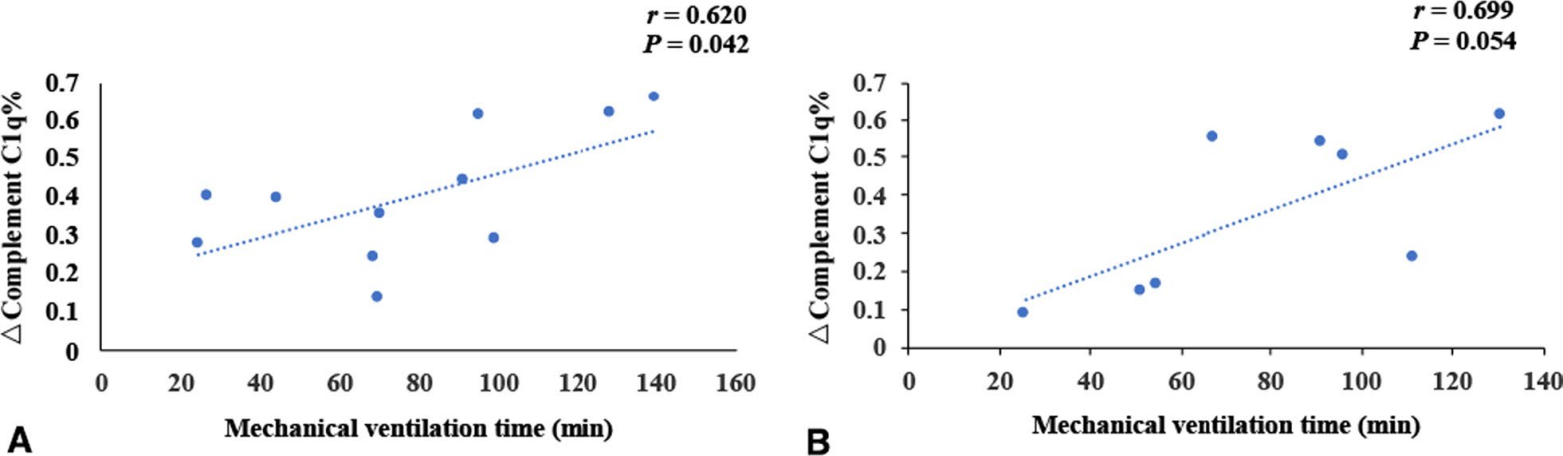
The correlation of mechanical ventilation time and the percentage decrease of complement C1q before and after operation in simple CoA group and Complex CoA group. (**A** shows the correlation of mechanical ventilation time and the percentage decrease of complement C1q before and after operation in simple CoA group (*n* = 11); **B** showed the correlation of mechanical ventilation time and the percentage decrease of complement C1q before and after operation in complex CoA group (*n* = 8). In simple CoA group, the percentage of postoperative decrease in C1q was positively correlated with the time of mechanical ventilation (*r* = 0.620, *P* < 0.05). However in complex CoA group, there was no significant correlation between the percentage of postoperative decrease in C1q and the time of mechanical ventilation (*r* = 0.699, *P* > 0.05).)

The percentage of postoperative decrease in C1q in children with simple CoA or complex CoA was positively correlated with the time of cardiopulmonary bypass and aortic cross clamp, respectively (*r* = 0.797, *r* = 0.622, *r* = 0.898, *r* = 0.920, *P* < 0.05) (Table 5, Fig. 5–6). In simple CoA group, the percentage of postoperative decrease in C1q was positively correlated with the time of mechanical ventilation (r = 0.620, *P* < 0.05) (Table 5, Fig. 7). But in complex CoA group, there was no significant correlation between the percentage of postoperative decrease in C1q and the time of mechanical ventilation (*r* = 0.699, *P* > 0.05) (Table 5, Fig. 7).

## Discussion

Congenital coarctation of the aorta (CoA) is a kind of localized aortic stenosis near the arterial catheter that was first described by Morgagni in 1760 [18]. CoA can be divided into simple CoA and complex CoA according to whether it is accompanied by important intracardiac pathological changes. Simple CoA refers to CoA with or without patent ductus arteriosus (PDA), while complex CoA refers to CoA with important intracardiac pathological changes, including ventricular septal defect (VSD), atrial septal defect (ASD), aortic valve stenosis, and double outlet of the right ventricle. The most common combined intracardiac malformation is VSD [5]. The hemodynamic changes in CoA are closely related to the severity of the coarctation and the combined intracardiac malformations. The main hemodynamic changes caused by CoA include upper limb hypertension and left ventricular hypertrophy. The increase in blood flow above the coarctation and the ejection resistance of the left ventricle increase the blood pressure of the upper limb, while the decrease in blood flow below coarctation leads to a decrease in the blood pressure of the lower limb. At the same time, the main response of the left ventricle to outflow tract obstruction is compensatory left ventricular hypertrophy. Over time, continuous hypertension can further aggravate the left ventricular hypertrophy and myocardial fibrosis, resulting in reduced left ventricular compliance and diastolic heart failure. If not treated in time, it can progress to total heart failure.

The mechanisms of cardiac hypertrophy include cellular metabolism, proliferation, noncoding RNAs, immune responses, translational regulation, and epigenetic modifications [19]. Immune cell infiltration has been shown to be involved in the pathogenesis of CoA [20]. So, in the left ventricular hypertrophy induced by congenital CoA, whether immune related factors are involved? To date, no studies have been reported.

The complement system is an important part of innate and adaptive immunity. Under physiological conditions, the activation and regulation of the complement system are in a balanced state. When the balance is broken, the complement system will attack the body’s own cells and tissues, which will lead to a variety of inflammatory reactions and autoimmune diseases [21]. C1q is an important promoter of the classical complement pathway. It activates the complement cascade reaction by recognizing the complement binding site of the antibody FC segment in an IgG or IgM immune complex, triggering the clearance of the antigen antibody complex [4]. It has been reported that complement C1q can lead to left ventricular hypertrophy by promoting *β*-catenin activation [22]. The C1q complement/TNF-related protein (CTRP) family has a spherical region at the C-end of the protein, its structure is similar to that of the complement component C1q, and there is a signal peptide at the N-end of the protein [23]. In 2019, Zhang B et al. reported that CTRP-3 could attenuate pressure overload-induced cardiac hypertrophy by suppressing the p38/CREB pathway and p38-induced ER stress [14]. Therefore, whether complement C1q is involved in the pathogenesis of congenital CoA and the regulation of left ventricular hypertrophy caused by CoA has not been reported in the literature.

Based on our study, we found that the content of complement C1q in the plasma of patients with simple CoA was significantly lower, and their IVSTZ and LVPWTZ were significantly higher. By further analyzing the relationship between complement C1q and left ventricular hypertrophy, we found that the content of complement C1q before the operation was negatively correlated with IVSTZ and LVPWTZ. These data indicate that complement C1q has a certain inhibitory effect on the formation of left ventricular hypertrophy. Meanwhile, we also found that the IVSTZ and LVPWTZ were not significantly different between the complex CoA group and the large VSD group, which may be related to the large left to right shunts at the ventricular level, but the LVDdZ in the complex CoA group was significantly lower than that in the large VSD group. We think that this may be related to the reduction in the left to right shunt flow through the ventricular septum, which was induced by the obstruction of the left ventricular outflow tract caused by CoA.

During cardiac surgery with cardiopulmonary bypass and aortic cross clamp, the heart is isolated from the circulation. This inevitably induces myocardial ischemia. In addition to this ischemic insult, an additional hit will occur upon reperfusion, which may worsen the extent of tissue damage and organ dysfunction [24]. In 2020, Miyamoto T found that in pediatric cardiac surgery with cardiopulmonary bypass, the C1q concentration was significantly lower. For patients who were given C1 esterase inhibitors intravenously 60 min after CPB, the classical complement pathway was activated through the replenishment of complement [25].

Mechanical ventilation can also promote the release of inflammatory mediators and activate the inflammatory response. Silvia Marchesi et al. reported that the blood IL6 and TNF-α ratio increased significantly in patients on mechanical ventilation [26]. Dhanireddy S found that mechanical ventilation could exacerbate both pulmonary and systemic inflammation in response to bacteria and contribute to the pathogenesis of both acute lung injury and multiple organ dysfunction syndromes, it could augment pulmonary production of the proinflammatory cytokines KC, MIP-2, TNF-alpha, and IL-6 and increase the alveolar-capillary permeability to proteins [27].

By analyzing complement C1q in children with CoA before and 24 h after the operation, we found that the decreased percentage of C1q in children with simple CoA or complex CoA was positively correlated with the time of cardiopulmonary bypass and aortic cross clamp. In the simple CoA group, the percentage of postoperative decrease in C1q was positively correlated with the time of mechanical ventilation. However, in the complex CoA group, there was no significant correlation between the percentage of postoperative decrease in C1q and the time of mechanical ventilation. These results show that with the extension of cardiopulmonary bypass time and aortic cross clamp time, complement C1q is gradually consumed and reduced. Complement C1q may have a certain protective effect on myocardial ischemia reperfusion during cardiopulmonary bypass and aortic cross clamp. In terms of mechanical ventilation time, with the extension of mechanical ventilation time, complement C1q gradually decreases, which may be due to mechanical ventilation causing inflammatory reactions, and complement C1q protects other organs and tissues by inhibiting inflammatory reactions. The continuous activation and consumption of complement C1q in this process eventually leads to a gradual decrease in complement C1q.

Abnormal lipid metabolism of cardiomyocytes can cause left ventricular hypertrophy. Celentano A found that hypercholesterolemia in normotensive nondiabetic adults is independently associated with a mildly concentric left ventricular geometry [28]. Increased left ventricular afterload leads to myocyte hypertrophy and interstitial fibrosis, which has been shown to cause concentric left ventricular hypertrophy [29]. The energy supply of cardiomyocytes mainly depends on the oxidation of long-chain fatty acids, producing ATP through mitochondria, while the obstruction of fatty acid oxidation and the enhancement of the myocardial glucose metabolism pathway are related to myocardial hypertrophy [30]. Therefore, there is a close relationship between lipid metabolism and left ventricular hypertrophy.

In 2004, Wong GM et al. identified C1q complement/TNF-related protein (CTRP) [23]. To date, the literature has reported that there are 15 CTRP family members (CTRP 1–15). The CTRP protein family has a spherical region at the C-end of the protein and its structure is similar to that of the complement component C1q. There is a signal peptide at the N-end of the CTRP protein. Serum CTRP levels are negatively correlated with TCHO and TG and positively correlated with high-density lipoprotein cholesterol (HDL-C) [31, 32]. Complement C1q and CTRP partially share the same structure, but whether complement C1q is related to lipid metabolism in patients with CoA has not been reported in the literature. Through this study, we found that there was no significant difference in plasma TG, TCHO, HDL-C or LDL-C between CoA children and children without an increased left ventricular afterload before operation, and there was no correlation between the complement C1q level and lipid metabolism-related indicators before operation. This may be because the children we included in this study were young and the course of the disease was short, so they did not develop abnormal lipid metabolism. Additionally, complement C1q may not inhibit the formation of left ventricular hypertrophy by regulating lipid metabolism in CoA-induced left ventricular hypertrophy. After operation, the complement C1q was positively correlated with TCHO, HDL and LDL in complex CoA group, but there was no correlation between the complement C1q level and TG. This may be related to the effect of surgical trauma on lipid metabolism. This mechanism needs further study.

In conclusion, in this study, by analyzing the content of complement C1q in the plasma of children with simple and complex CoA and the relationship between the content of complement C1q and LVPW, IVS and LVDd, we found that complement C1q has an inhibitory effect on CoA-induced left ventricular hypertrophy, which is negatively correlated with left ventricular myocardial hypertrophy. By analyzing the content of complement C1q in children with CoA before and 24 h after the operation, it was found that the decreased percentage of complement C1q after the operation was positively correlated with the time of cardiopulmonary bypass, aortic cross clamp and mechanical ventilation. We also found that there was no significant correlation between complement C1q and related indicators of lipid metabolism. The above results have certain clinical significance for clarifying the pathogenesis of left ventricular hypertrophy caused by congenital CoA and the role of complement C1q in myocardial injury during cardiac surgery.

There were some limitations in our study. First, this was a retrospective study, there was only the value of complement C1q at 24 h after operation, and the values at other times after operation were incomplete. Therefore, we only analyzed the complement C1q level at 24 h after the operation. The time of echocardiogram reexamination after operation was inconsistent, so the correlation between postoperative complement C1q and LVDd, IVST and LVPWT could not be analyzed. Second, the number of patients included in this study is small. In the future, we will conduct a large clinical prospective study to further investigate the correlation between complement C1q levels and left ventricular hypertrophy induced by coarctation of the aorta.

## Conclusions

C1q is an important promoter of the classical complement pathway. In our study, we found that serum complement C1q levels in simple CoA patients showed lower level. In CoA patients, serum complement C1q had a negative association with interventricular septal thickness and left ventricular posterior wall thickness. The decreasing of serum complement C1q was an unfavorable factor for acute myocardial injury during cardiac surgery. Complement C1q may be related to the process of left ventricular hypertrophy induced by CoA and have a protective effect against myocardial injury during cardiac surgery.

## Data Availability

All data generated or analyzed during this study are included in this published article.

## Abbreviations

CoA: Coarctation of the aorta
VSD: Ventricular septal defect
PDA: Patent ductus arteriosus
TG: Triglycerides
TCHO: Total cholesterol
HDL-C: High-density lipoprotein cholesterol
LDL-C: Low-density lipoprotein cholesterol
LVDd: Left ventricular end diastolic diameter
IVST: Interventricular septal thickness
LVPWT: Left ventricular posterior wall thickness
CTRP: C1q complement/TNF-related protein
